# The Rate of Sputum Smear-Positive Tuberculosis after Treatment Default in a High-Burden Setting: A Retrospective Cohort Study

**DOI:** 10.1371/journal.pone.0045724

**Published:** 2012-09-25

**Authors:** Florian M. Marx, Rory Dunbar, Donald A. Enarson, Nulda Beyers

**Affiliations:** 1 Charité – Universitätsmedizin, Department for Pediatric Pneumology and Immunology, Berlin, Germany; 2 Desmond Tutu TB Centre, Stellenbosch University, Tygerberg, South Africa; 3 International Union Against Tuberculosis and Lung Disease, Paris, France; McGill University, Canada

## Abstract

**Rationale:**

High rates of recurrent tuberculosis after successful treatment have been reported from different high burden settings in Sub-Saharan Africa. However, little is known about the rate of smear-positive tuberculosis after treatment default. In particular, it is not known whether or not treatment defaulters continue to be or become again smear-positive and thus pose a potential for transmission of infection to others.

**Objective:**

To investigate, in a high tuberculosis burden setting, the rate of re-treatment for smear-positive tuberculosis among cases defaulting from standardized treatment compared to successfully treated cases.

**Methods:**

Retrospective cohort study among smear-positive tuberculosis cases treated between 1996 and 2008 in two urban communities in Cape Town, South Africa. Episodes of re-treatment for smear-positive tuberculosis were ascertained via probabilistic record linkage. Survival analysis and Poisson regression were used to compare the rate of smear-positive tuberculosis after treatment default to that after successful treatment.

**Results:**

A total of 2,136 smear-positive tuberculosis cases were included in the study. After treatment default, the rate of re-treatment for smear-positive tuberculosis was 6.86 (95% confidence interval [CI]: 5.59–8.41) per 100 person-years compared to 2.09 (95% CI: 1.81–2.41) after cure (adjusted Hazard Ratio [aHR]: 3.97; 95% CI: 3.00–5.26). Among defaulters, the rate was inversely associated with treatment duration and sputum conversion prior to defaulting. Smear grade at start of the index treatment episode (Smear3+: aHR 1.61; 95%CI 1.11–2.33) was independently associated with smear-positive tuberculosis re-treatment, regardless of treatment outcome.

**Conclusions:**

In this high-burden setting, there is a high rate of subsequent smear-positive tuberculosis after treatment default. Treatment defaulters are therefore likely to contribute to the pool of infectious source cases in the community. Our findings underscore the importance of preventing treatment default, as a means of successful tuberculosis control in high-burden settings.

## Introduction

A major principle in tuberculosis control is the necessity to ensure that patients adhere to a full course of treatment. At least six months anti-tuberculosis multidrug chemotherapy is required to achieve cure in smear-positive tuberculosis patients with initially drug-susceptible disease [1]. Shorter treatment regimens result in high rates of disease recurrence within 24 months after treatment [2]–[4].

Adherence to a full course of treatment is therefore expected not only to prevent disease recurrence, but also to contribute to a reduction in tuberculosis burden at population level [5]. The latter is based on the assumption that patients not successfully treated remain contagious or experience recurrent disease and contribute to an increased burden of disease and transmission of tuberculosis within the community.

Non-adherence to a full course of anti-tuberculosis treatment is usually termed ‘treatment default’, defined as interruption of treatment for at least two consecutive months. Risk factors for treatment default such as lack of knowledge and family support, distance between home and health care facility, and drug side effects have been widely studied [6]–[8], but little is known about the fate of patients after defaulting from anti-tuberculosis treatment. It is not known whether or not treatment defaulters continue or return again sputum smear-positive and thus pose a potential for transmission of infection to others.

While treatment defaulters could be specifically targeted by interventions to prevent default, to retrieve those who have defaulted and to prevent subsequent recurrence of disease [9], such interventions require scarce resources that must be rationed properly based on an assessment of the size of the problem and the ease of its solution.

This study was conducted in a setting with a high tuberculosis burden in South Africa. High rates of recurrent tuberculosis after successful treatment have been reported from this and other settings in Sub-Saharan Africa. Exogenous re-infection rather than relapse seems to be the major underlying cause of recurrence in successfully treated cases [10]–[12], with the proportion of re-infection increasing with background tuberculosis incidence [13], and with human immunodeficiency virus (HIV) co-infection being an important risk factor [14]–[16].

In the context of frequent tuberculosis re-infection, little is known about the significance of treatment default as a risk factor for smear-positive tuberculosis.

The objective of this study was to investigate the rate of re-treatment for smear-positive tuberculosis, after defaulting from an initial treatment episode. We hypothesised that in a setting with a high tuberculosis burden, sputum smear-positive tuberculosis cases who default from treatment are more likely to return for treatment with smear-positive disease compared to those who successfully complete their treatment. We further aimed to investigate whether treatment duration and sputum conversion prior to default are associated with re-treatment for smear-positive tuberculosis.

**Figure 1:**
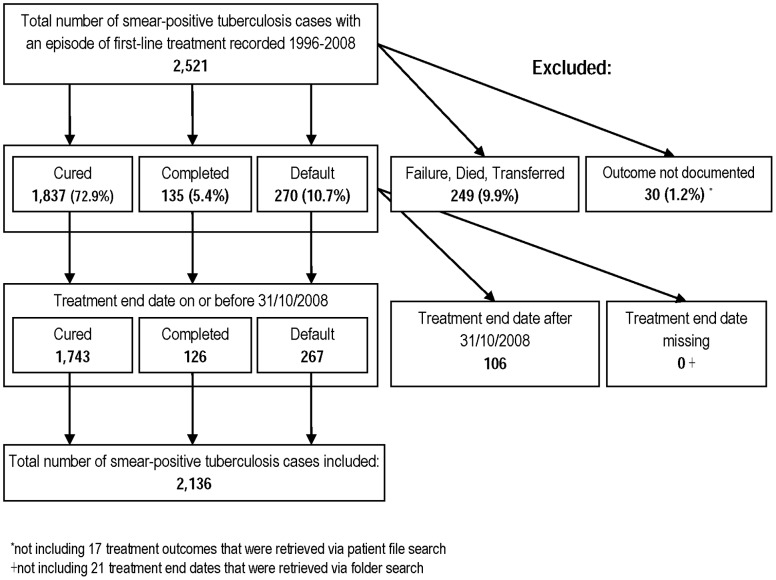
Overview of smear-positive tuberculosis cases included in and excluded from the study.

## Methods

### Study Setting

Two adjacent urban communities covering an area of 3.4 km^2^ with 36,000 inhabitants of low socio-economic status and a high-burden of tuberculosis in metropolitan Cape Town, South Africa [17]. The DOTS strategy [18] was introduced in 1996 to both communities where two primary health-care clinics provide treatment and routinely record and report cases started on treatment. Treatment regimens were in accordance with standards for South Africa [19]: For cases never previously treated (new cases), this was six months of daily isoniazid and rifampicin supplemented by daily pyrazinamide and ethambutol for the first two months, extended for a further month if the sputum smear was positive at the end of two months. Treatment for re-treatment cases consisted of daily isoniazid, rifampicin and ethambutol for eight months supplemented with daily pyrazinamide and streptomycin in the first three months.

**Figure 2:**
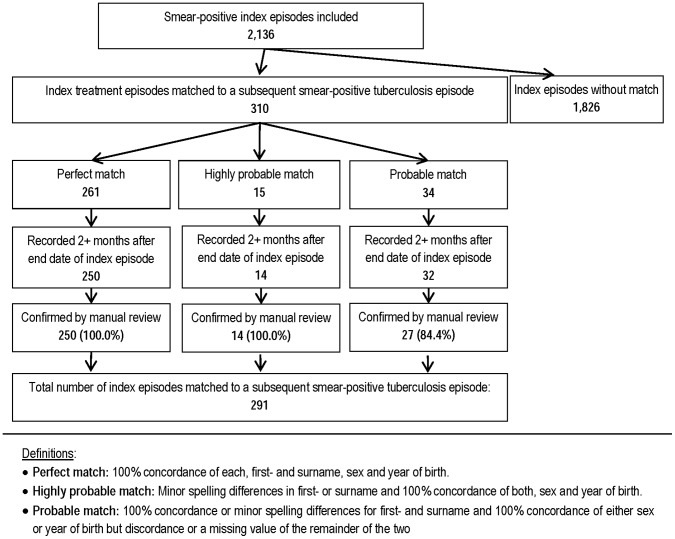
Ascertainment of subsequent episodes of re-treatment via record linkage and manual review for the study.

Treatment outcomes were documented by local health care staff according to standard definitions [19]. This included cure, for patients who were smear-positive at initiation of treatment and smear-negative at, or one month prior, to the completion of treatment and also on at least one previous occasion, and treatment completed, for patients who had completed treatment but without proof of cure due to smear results not available on at least two occasions prior to the completion of treatment. The term ‘treatment success’ includes both, cure and treatment completed. The outcome of treatment default was recorded for a tuberculosis case whose treatment was interrupted for more than two consecutive months before the end of the treatment period. Health care staff members were advised to record the date of treatment default as the last date at which the patient picked up medication before defaulting, and, in the case of interruption for less than two months, to trace the patient and to prolong treatment in order to compensate for missed doses.

**Table 1:** Univariable analysis of index episode risk factors for subsequent smear-positive tuberculosis re-treatment (N = 2,136).

	Total re-treatmentcases	PY	Rate (per 100 PY)	Crude hazard-ratio(95% CI)	P-value
**Overall**	291	10844	2.68 (2.39–3.01)		
**Treatment outcome**					<0.001
Cured	185	8863	2.09 (1.81–2.41)	1	
Completed	14	640	2.19 (1.29–3.69)	1.05 (0.61–1.80)	
Defaulted	92	1341	6.86 (5.59–8.41)	3.29 (2.56–4.22)	
**Sex**					0.005
Female	93	4352	2.14 (1.74–2.62)	1	
Male	198	6492	3.05 (2.65–3.51)	1.43 (1.12–1.83)	
**Age**					0.002
0–18	30	758	3.96 (2.77–5.66)	1	
19–39	193	6646	2.90 (2.52–3.34)	0.73 (0.50–1.08)	
40+	68	3430	1.98 (1.56–2.51)	0.50 (0.33–0.77)	
**Patient category**					0.03
New	181	7384	2.45 (2.12–2.84)	1	
Re-treatment	110	3424	3.21 (2.67–3.87)	1.31 (1.03–1.66)	
**HIV status**					0.10
negative	111	3542	3.13 (2.60–3.77)	1	
positive	12	383	3.13 (1.78–5.52)	1.00 (0.55–1.81)	
unknown	168	6919	2.43 (2.09–2.82)	0.77 (0.61–0.98)	
**Smear grade** *					0.03†
Smear 1+	35	1588	2.20 (1.58–3.07)	1	
Smear 2+	48	1576	3.05 (2.30–4.04)	1.38 (0.89–2.14)	
Smear 3+	154	4641	3.32 (2.83–3.89)	1.51 (1.04–2.17)	
**Smear conversion (month 2)**					0.03
Yes (smear-negative)	189	8297	2.28 (1.98–2.63)	1	
No (smear-positive)	60	1899	3.16 (2.45–4.07)	1.39 (1.04–1.85)	
**Treatment duration**					<0.001
>8 months	53	1858	2.85 (2.18–11.32)	1.47 (1.06–2.03)	
6–8 months	120	6177	1.94 (1.62–2.32)	1	
4 – <6 months	88	2431	3.62 (2.94. –4.46)	1.86 (1.42–2.45)	
<4 months	30	379	7.92 (5.54–12.23)	4.08 (2.73–6.08)	

PY = Person-years.

CI = Confidence Interval.

* Smear grade at start of treatment:

Smear 3+: Any of the two initial smears was 3+,i.e. >10 acid-fast bacilli (AFB) per 1 high-power field (HPF).

Smear 2+: Any of the two initial smears was 2+,i.e. 1–10 AFB per 1 HPF, but none of them was 3+.

Smear 1+: Any of the two initial smears was 1+,i.e. 10–99 AFB per 100 HPF, but none of them was 2+ or 3+.

† Test for trend.

**Table 2:** Multivariable analysis of index episode risk factors for subsequent smear-positive tuberculosis re-treatment (N = 1,733).

	Total re-treatment cases	PY	Rate (per 100 PY)	Crude hazard-ratio* (95% CI)	P-value
**Treatment outcome**					<0.001
Cured	185	8863	2.09 (1.81–2.41)	1	
Completed	14	640	2.19 (1.29–3.69)	1.08 (0.58–2.00)	
Defaulted	92	1341	6.86 (5.59–8.41)	3.97 (3.00–5.26)	
**Smear grade** †					0.03‡
Smear 1+	35	1588	2.20 (1.58–3.07)	1	
Smear 2+	48	1576	3.05 (2.30–4.04)	1.43 (0.93–2.22)	
Smear 3+	154	4641	3.32 (2.83–3.89)	1.61 (1.11–2.33)	
**Age**					0.003
0–18	30	758	3.96 (2.77–5.66)	1	
19–39	193	6646	2.90 (2.52–3.34)	0.50 (0.32–0.78)	
40+	68	3430	1.98 (1.56–2.51)	0.40 (0.25–0.65)	

PY = Person-years.

CI = Confidence Interval.

* Adjusted for the other factors shown in the table.

† Smear grade at start of treatment:

Smear 3+: Any of the two initial smears was 3+, i.e. >10 acid-fast bacilli (AFB) per 1 high-power field (HPF).

Smear 2+: Any of the two initial smears was 2+, i.e. 1–10 AFB per 1 HPF, but none of them was 3+.

Smear 1+: Any of the two initial smears was 1+, i.e. 10–99 AFB per 100 HPF, but none of them was 2+ or 3+.

‡ Test for trend.

### Data Sources

Routine program data entered by local health care staff from both clinics into paper-based tuberculosis treatment registers for the years 1996 to 2008 were captured in an electronic database. Missing values for treatment end date and treatment outcome were ascertained as follows: If no end date (default date) was documented in the treatment register, the patient file was reviewed and the last day at which the patient was documented to have taken up medication was accepted as the end date. If no treatment outcome was documented in the register, patient files were reviewed and the documented treatment outcome was updated in the database.

**Figure 3:**
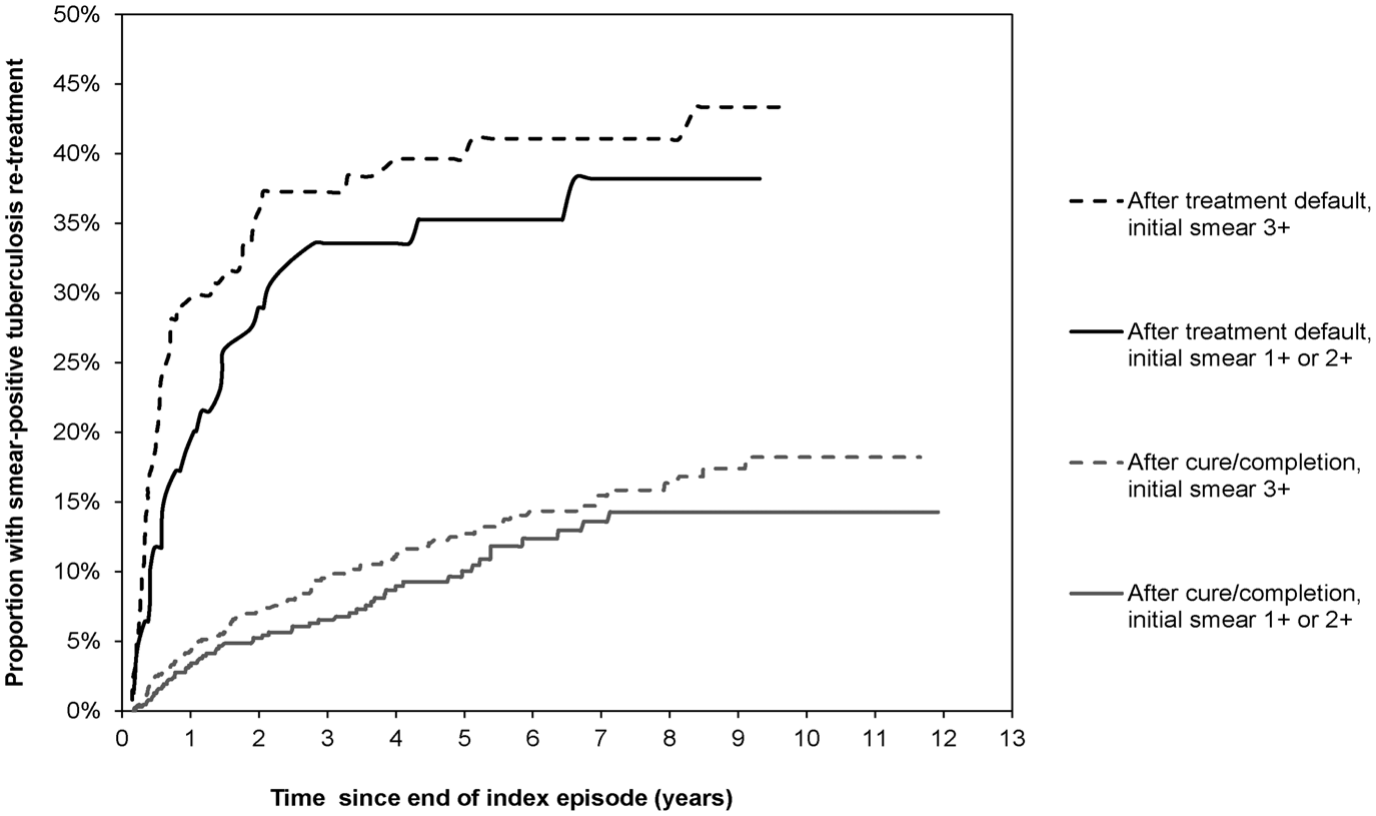
Kaplan Meier failure estimates of re-treatment for smear-positive tuberculosis by index episode sputum smear grading and treatment outcome.

### Study Design

A retrospective cohort study was conducted, comparing sputum smear-positive tuberculosis cases who successfully completed treatment to those who defaulted from treatment between 1996 and 2008. From the database, we selected all episodes of treatment with a documented smear-positive sputum result at start of treatment and a treatment outcome of either cure, treatment completed or treatment default. ‘Sputum smear-positive’ was defined by at least one sputum sample documented smear-positive for acid-fast bacilli by fluorescence microscopy. Episodes of second-line treatment, episodes of treatment for smear-negative tuberculosis, episodes with treatment outcomes other than success or default and those without a treatment outcome documented, were not included in the study. We further excluded episodes with a treatment end date after 31^st^ October 2008, in order to allow for sufficient time to re-treatment (see below).

**Figure 4:**
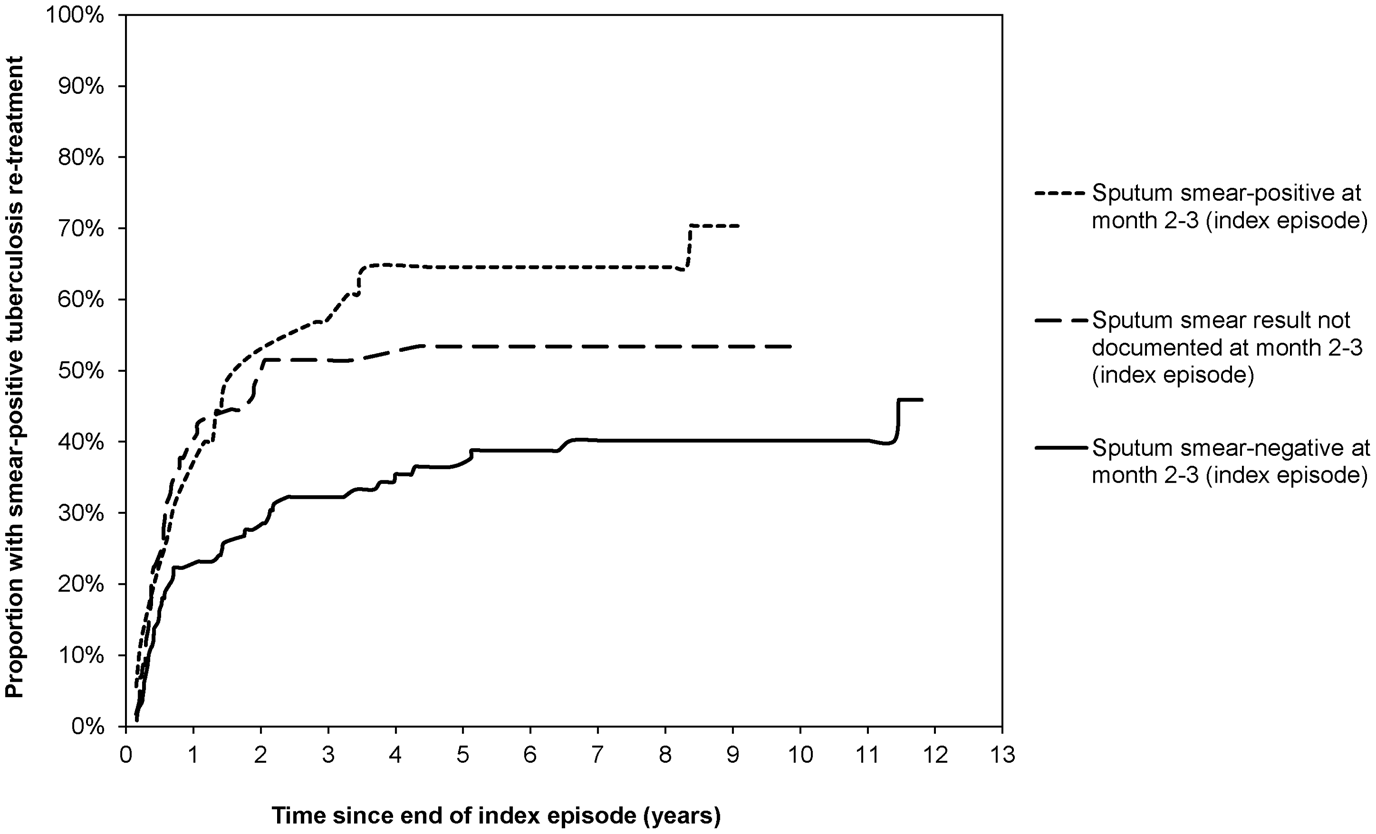
Kaplan Meier failure estimates of smear-positive re-treatment after treatment default, stratified by smear conversion prior to defaulting (adjusted for time to default).

All treatment episodes included in the study were defined as index episodes. An episode of re-treatment for smear-positive tuberculosis (study outcome) was defined as any subsequent episode of treatment for documented smear-positive tuberculosis recorded for the same individual person, with a minimum time of two months between the end date of the index episode and the record date of the re-treatment episode.

### Ascertainment of Smear-positive Tuberculosis Re-treatment

We used Registry Plus™/Link-Plus [20] probabilistic record linkage software to identify episodes of re-treatment for smear-positive tuberculosis recorded in the treatment registers. The software matches records on the basis of matching variables and assigns a probability score for a true (individual) match of two independent records. Each index treatment episode included in the study was screened for matches with any subsequent treatment episode recorded for the same individual person in the treatment registers. First and family name (case-sensitive), sex and year of birth were chosen as matching variables.

**Table 3:** Treatment outcomes at re-treatment stratified by index episode treatment outcome (N = 291 re-treatment cases).

		Treatment outcome, re-treatment episode					
	Total	Cured/Completed	Failed	Died	Defaulted	Transfer out	Unknown
**Treatment outcome, index episode**							
Cured/Completed	199 (100.0%)	152 (76.4%)	12 (6.0%)	8 (4.0%)	17 (8.5%)	2 (1.0%)	8 (4.0%)
Treatment default	92 (100.0%)	49 (53.3%)	3 (3.3%)	2 (2.2%)	34 (37.0%)	3 (3.3%)	1 (1.1%)
**Total**	**291 (100.0%)**	**201 (69.1%)**	**15 (5.2%)**	**10 (3.4%)**	**51 (17.5%)**	**5 (1.7%)**	**9 (3.1%)**

Matches of treatment episodes identified from the record linkage were initially reviewed and classified on the basis of concordance of the matching variables into perfect matches, highly probable matches and probable matches: A ‘perfect match’ was defined by 100% concordance of each, first and surname, sex and year of birth. ‘Highly probable matches’ had minor spelling differences in first- or surname and 100% concordance of both, sex and year of birth. ‘Probable matches’ had 100% concordance or minor spelling differences of first- and surname and 100% concordance of either sex or year of birth but discordance or a missing value of the remainder of the two. Matches identified by the software that did not meet these criteria were discarded.

In order to ensure that index and re-treatment episode belonged to the same individual person, all perfect, highly-probable and probable matches underwent careful manual review using the original paper-based treatment registers and, if necessary, patient folders as a reference. In line with the study definition for episodes of re-treatment for smear-positive tuberculosis, matches were excluded if the time period between the end date of the index episode and the record date of the re-treatment episode was shorter than two months.

### Data Analysis

STATA™ 10.1 statistical application (Stata Corp, College Station, TX, USA) was used for data analysis. Survival analysis and Poisson regression were used to determine rates of re-treatment for smear-positive tuberculosis after treatment default vs. after cure/completion. Cases entered the study on the date when they successfully completed or defaulted from the index treatment episode and were censored on the date when the first re-treatment episode (smear-positive) was recorded in the registers or else at the end of the study period. A multivariable regression model was developed using a step-wise forward technique: co-factors documented at the index episode were considered if they reached P<0.10 significance at univariable regression and resulted in a change of +/−0.1 Rate Ratio for the principal determinant under study. Among treatment defaulters, sub-group analysis was conducted, taking into consideration treatment duration, and sputum conversion prior to defaulting, as documented at the end of the intensive phase of treatment (month 2–3).

### Ethics Statement

Permission to access the program data for research has been granted by the City of Cape Town (research ID = 10142), the custodian of the data. The study was approved by the Committee for Human Research, Faculty of Health Sciences, Stellenbosch University (N09/05/144 and amendments 1 and 3) and also by the Ethics Advisory Group of the International Union Against Tuberculosis and Lung Disease. This was a retrospective analysis of routine data and therefore we requested and were granted a waiver of individual informed consent from the ethics committee. The use of a unique subject ID ensured anonymous analysis. Only the senior data staff had access to personal identifiers which were removed after the record linkage process.

## Results

A total of 2,521 tuberculosis cases with an episode of first-line treatment for smear-positive tuberculosis were recorded in the treatment registers 1996–2008. Of these, 2,136 were included in the study, 1,743 with documented cure, 126 with treatment completion and 267 with treatment default. A breakdown of cases included and excluded is shown in Figure 1.

Of all cases included, 1,274 (59.6%) were male, median age was 34 years, and 1,065 (49.9%) had an HIV test result documented, 110 (10.3%) of whom were HIV positive.

### Re-treatment for Smear-positive Tuberculosis

For 291 (13.6%) of the 2,136 smear-positive cases included in the study, an episode of re-treatment for smear-positive tuberculosis was identified via record linkage and confirmed via manual review. A detailed overview of the record linkage and manual review is shown in Figure 2.

Median time between the end date of the index episode and the record date of the subsequent episode was 17 months. Ninety-two of the 291 cases with re-treatment for smear-positive tuberculosis (31.6%) had defaulted at the index treatment episode.

The rate of re-treatment for smear-positive tuberculosis was 2.09 (95% confidence interval [CI]: 1.81–2.41) per 100 person-years after cure, 2.19 (95%CI: 1.29–3.69) after treatment completion, and 6.86 (95%CI: 5.59–8.41) after previous treatment default.

For tuberculosis cases defaulting during the index episode, the unadjusted Hazard Ratio (HR) of re-treatment was 3.29 (95%CI: 2.56–4.22) using cases with cure as the baseline (Table 1). Sputum smear grading at start of the index treatment episode (Smear 3+: HR 1.61; 95%CI: 1.11–2.33) and age (≥40 years: HR 0.40; 95%CI: 0.25–0.65) were each independently associated with smear-positive tuberculosis re-treatment (Table 2). After adjusting for initial smear grade and age, the HR for smear-positive tuberculosis re-treatment among defaulters was 3.97 (95%CI: 3.00–5.26).

In cases after treatment default, there was a steep increase in the cumulative rate of smear-positive tuberculosis within the first two years. By the end of the second year, 27.9% (95% CI 22.8% –33.8%) had experienced a re-treatment episode, compared to 5.8% (95%CI: 4.7%–7.0%) of cases after cure. The failure function for cases after default differed according to smear-grading at start of treatment (P = 0.04; after treatment success: P = 0.05) (Figure 3).

### Re-treatment Rates by Treatment Duration and Smear Status Prior to Defaulting

Among the 267 treatment defaulters, 17 (6.4%) defaulted during the intensive phase of treatment. There was an inverse linear association between the time to treatment default (i.e. months of treatment prior to defaulting) and the rate of re-treatment (aHR: 0.87 [95%CI: 0.78–0.97] per one month of treatment; P = 0.01).

Of the treatment defaulters, the sputum smear result at the end of the intensive phase of treatment was negative in 149 (55.8%), positive in 43 (16.1%) and not documented in 75 (28.1%). The rate of smear-positive tuberculosis re-treatment was 5.81 (95% CI: 4.34–7.78) per 100 person-years among defaulters with documented sputum conversion prior to defaulting, 6.07 (95% CI: 3.66–10.07) with documented positive sputum smear result at follow-up, and 10.00 (95% CI: 7.07–14.14) among treatment defaulters without documented follow-up sputum smear-result. Figure 4 shows the failure function for smear-positive tuberculosis re-treatment by sputum smear status prior to defaulting, adjusted for time to treatment default.

### Treatment Outcomes at Re-treatment

Thirty-four (37.0%) of the 92 re-treatment tuberculosis cases who defaulted from treatment at the index episode, defaulted again from re-treatment, compared to 17 (8.5%) of 199 re-treatment cases defaulting after previous cure or treatment completion (Table 3).

## Discussion

This study demonstrates for the first time the substantially high rate of smear-positive tuberculosis associated with previous treatment default. Treatment default remains a major risk factor for subsequent smear-positive tuberculosis, even in settings where tuberculosis recurrence due to re-infection is generally common.

The rates of smear-positive tuberculosis presented here are based on treatment records and likely underestimate the true rates of smear-positive tuberculosis after cure, treatment completion and default, because some of the tuberculosis cases might not have returned for treatment or they might have sought treatment elsewhere, and because the population at risk in this study includes those who might have moved or died.

The differences in the rates of smear-positive tuberculosis after treatment default vs. after success are due to the strikingly higher rates within the first two years in those defaulting from treatment. Our results suggest that tuberculosis had been initially contained in the majority of treatment defaulters, i.e. those with longer time to default and with documented smear conversion, and worsened very soon after stopping the treatment. Cases defaulting earlier from treatment and those who continue smear-positive most likely continue to suffer from active disease resulting in high rates of re-treatment within the first two years after defaulting. Absence of smear examination recorded at the end of the initial phase may be a marker of irregular clinic attendance prior to default.

Treatment default is widely considered a risk factor for disease re-activation, but very few studies have looked at rates of tuberculosis after treatment default. A study on re-infection among successfully treated cases in the same communities 1993–1998 documented the frequency of culture confirmed tuberculosis in a sample of previous treatment defaulters for whom strain type information was available, similar to those we found [11]. A study conducted by Vree et al. in rural Vietnam found of 33 treatment defaulters with known outcome, seven had died; only one was found to be culture positive and 23 smear- and culture negative [21].

The rate of recurrent smear-positive tuberculosis after treatment success corresponds well with previous findings from the Western Cape Province [11] and with reports from late phase clinical trials with a rate of 5.0% at 24 months of follow-up in 282 cases treated in routine public services in Benin, Guinea, Tanzania, Mozambique, Nepal and China, and a rate of 3.8% at 18 months of follow-up in 1,103 cases treated in Guinea, Tanzania, Mozambique, Algeria, Nepal, Vietnam, Bolivia, Colombia and Peru [22], [23].

The more constant rate of recurrent episodes after treatment success suggests that recurrence in this group may be more likely due to re-infection, as suggested previously [11], [12].

The extent to which smear-positive cases after both, previous default and previous success, maintain the tuberculosis epidemic in this high-burden community cannot be determined from the results of this study. Den Boon et al. previously showed in a prevalence survey conducted in the same setting, that 56% of previously undetected prevalent smear-positive tuberculosis cases had a history of previous treatment [24]. These findings along with the findings of our study support the hypothesis that previously treated cases contribute considerably to the pool of infectious cases in the community.

Although the rate of smear-positive tuberculosis among treatment defaulters is higher, cases after treatment success account for the vast majority of recurrent smear-positive tuberculosis in this setting, a finding that is similar to those reported by Wood et al. in a study using notification data from Cape Town [25]. This is explained by a higher absolute number of successfully treated cases, and a rate of recurrence in this group that is lower in the first two years but constant in the following years.

We did not find evidence in our study that the rate of smear-positive recurrent tuberculosis was higher among HIV co-infected cases. HIV infected individuals may be less likely to be smear-positive upon recurrence and those living with HIV might be more likely to die, reducing the ‘person-years at risk’ which would result in lower rates.

Sputum smear grade at start of treatment was associated with smear-positive recurrence, independent of the later treatment outcome – similar to findings reported by Hesseling et al. in a cohort study from the same community using 24 months follow-up [26]. Tuberculosis cases with a high initial yield of mycobacteria might represent those with more severe, i.e. cavitating, disease [27].

Our study has limitations. We made use of probability record linkage in order to identify subsequent episodes of treatment among individuals. Although this method is considered sensitive [28], we might have failed to detect re-treatment episodes of smear-positive tuberculosis in some patients, if for instance women married and changed their surname. Further, the design of our study did not allow us to capture events of subsequent smear-positive tuberculosis untreated or treated elsewhere. The ‘population at risk’ might have changed according to individuals who died or who moved away from the area. All these factors would have led ultimately to an underestimate of the rates of smear-positive tuberculosis. Rates would have been overestimated if the linkage of episodes was less specific. However, we tried to exclude this possibility by using clear definitions of matches and conducting a careful manual review. We are therefore confident that the rates presented here were unaffected by matching error.

Residual confounding might have occurred in our study, given that we were unable to control for other factors known to affect the risk of tuberculosis recurrence, such as smoking, persistent cavities, or undetected drug-resistant disease [29]–[32].

Further, this study is an evaluation of routine health services based on the information that existed within those services. Such information might be less accurate than information prospectively collected as part of a research project. As noted above, the study did not take into account patients who, for whatever reason, did not return to the health service.

### Conclusions

We show for the first time that in a high-burden setting, tuberculosis cases defaulting from their treatment are at high risk of subsequent smear-positive disease. They may thus very likely experience adverse health effects including chronic pulmonary impairment [33], death [34], and acquisition of drug-resistance [35]. Further, they may contribute to the pool of infectious source cases in the community. Moreover, previous defaulters in this setting are at high risk of defaulting again from treatment. There is an urgent need to enhance treatment adherence in order to avoid these untoward events.

Further research is needed to understand to what extent treatment defaulters contribute to overall transmission of tuberculosis within high-burden communities and whether preventing default is an efficient means for reducing tuberculosis transmission. Further, it needs to be determined whether treatment default contributes to the acquisition and transmission of drug resistance in high-burden communities.
