# Correction: The Rate of Sputum Smear-Positive Tuberculosis after Treatment Default in a High-Burden Setting: A Retrospective Cohort Study

**DOI:** 10.1371/annotation/ea5f2a13-4394-41af-84c8-3e6af4a07770

**Published:** 2013-08-06

**Authors:** Florian M. Marx, Rory Dunbar, Donald A. Enarson, Nulda Beyers

The affiliation "Desmond Tutu TB Centre, Stellenbosch University, Tygerberg, South Africa" is missing information. The correct affiliation is as follows:

Desmond Tutu TB Centre, Department of Paediatrics and Child Health, Stellenbosch University, Tygerberg, South Africa 

